#  Variations in lignin monomer contents and stable hydrogen isotope ratios in methoxy groups during the biodegradation of garden biomass

**DOI:** 10.1038/s41598-022-12689-1

**Published:** 2022-05-24

**Authors:** Qiangqiang Lu, Lili Jia, Mukesh Kumar Awasthi, Guanghua Jing, Yabo Wang, Liyan He, Ning Zhao, Zhikun Chen, Zhao Zhang, Xinwei Shi

**Affiliations:** 1 Key Laboratory of Soil Resource and Biotech Applications, Shaanxi Academy of Sciences, Shaanxi Engineering Research Centre for Conservation and Utilization of Botanical Resources, Xi’an Botanical Garden of Shaanxi Province (Institute of Botany of Shaanxi Province), No.17, Cuihua South Road, Xi’an, 710061 China; 2 School of Geography and Tourism, Shaanxi Normal University, Xi’an, 710119 China; 3 College of Forestry, Northwest A&F University, Yangling, 712100 China; 4 College of Natural Resources and Environment, Northwest A&F University, Yangling, 712100 China

**Keywords:** Biochemistry, Biotechnology, Chemical biology, Microbiology

## Abstract

Lignin, a highly polymerized organic component of plant cells, is one of the most difficult aromatic substances to degrade. Selective biodegradation under mild conditions is a promising method, but the dynamic variations in lignin monomers during the biodegradation of lignocellulose are not fully understood. In this study, we evaluated the differences in lignin degradation under different microbial inoculation based on the lignin monomer content, monomer ratio, and stable hydrogen isotope ratio of lignin methoxy groups (δ^2^H_LM_). The weight loss during degradation and the net loss of lignocellulosic components improved dramatically with fungal inoculation. Syringyl monolignol (S-lignin), which contains two methoxy groups, was more difficult to degrade than guaiacyl (G-lignin), which contains only one methoxy group. The co-culture of *Pseudomonas mandelii* and *Aspergillus fumigatus* produced the greatest decrease in the G/S ratio, but δ^2^H_LM_ values did not differ significantly among the three biodegradation experiments, although the enrichment was done within the fungal inoculation. The fluctuation of δ^2^H_LM_ values during the initial phase of biodegradation may be related to the loss of pectic polysaccharides (another methoxy donor), which mainly originate from fallen leaves. Overall, the relative δ^2^H_LM_ signals were preserved despite decreasing G/S ratios in the three degradation systems. Nevertheless, some details of lignin δ^2^H_LM_ as a biomarker for biogeochemical cycles need to be explored further.

With the rapid worldwide expansion of urbanization, garden biomass is becoming a major component of organic solid wastes^1^. However, its unrestrained disposal would waste a potentially important resource (i.e., organic matter) and potentially even become a source of environmental pollution^2^. Biodegradation, including composting, has garnered particular attention as a way to dispose of this biomass while efficiently recovering chemicals and nutrients that have commercial applications or ecological value^3,4^. Researchers have shown that biodegradation of this biomass under controlled or natural conditions can successfully decompose lignocellulosic material and allow the production of potentially useful alternative organic products by multiple microbial enzymes acting through different metabolic pathways^5,6^. Nevertheless, depolymerization of lignin is a particular challenge during bioconversion due to the relative hydrophobicity of macromolecular polymers and the antibacterial properties of aromatic structures^7,8^.

Lignin, the second-most abundant biomacromolecule, is predominantly composed of three 4-hydroxyphenylpropanoid units: guaiacyl monolignols (G-lignin), syringyl monolignols (S-lignin), and minor amounts of *p*-hydroxyphenyl monolignols (H-lignin), with the corresponding proportions varying with plant and tissue type. In plants, these aromatic monomers are highly interconnected through aryl ether, biphenyl ether, resinol, phenyl-coumaran, and diphenyl bonds, thus enhancing the strength and rigidity of these molecules, which makes them more difficult for microbial enzymes and chemical hydrolysis to degrade^9^. In addition, because the components of lignin have multiple cleavage sites for enzymatic hydrolysis and high oxidation potential for non-specific free radicals, biodegradation is still an efficient, cost-effective, and eco-friendly approach for lignin depolymerization^10,11^. The complex biochemical process of enzymatic hydrolysis of lignin begins with deconstruction to form heterogeneous aromatic hydrocarbons, which are then consumed by the central carbon metabolism^12^. In general, these ligninolytic enzymes (extracellular oxidases), which include laccase, manganese peroxidase, and lignin peroxidase, are mainly secreted by fungi and some bacteria^13,14^. Most research has focused on screening to identify lignin-degrading microorganisms and to characterize the expression of multienzyme activities; however, studies on the efficiency of lignin degradation by different microorganisms have been neglected^15^.

Previous studies of the chemical transformations that occur during lignin depolymerization have indicated that monolignol S-lignin, with two methoxy (OCH_3_) groups at the meta-positions (positions 3 and 5) of the phenylpropanoid structure, is more difficult to depolymerize than G-lignin (with a single OCH_3_ group) and H-lignin (with no OCH_3_ groups)^16–18^. This is mainly due to the irreversible methylation of coniferyl and sinapyl alcohols (3/5-O-methylation) via caffeic acid *O*-methyltransferases, caffeoyl-CoA *O*-methyltransferases, or both, which is different from the isomerization of aromatic hydrocarbons and the bonding of the propane side chains during monolignol biosynthesis^9^. Therefore, these stable OCH_3_ groups are not only chemical markers of structural differences in lignin monomers but also notable precursors of numerous organic compounds^19^. To date, lignin methoxy groups have been widely used in research on isotopic biogeochemistry, eco-climatology, and authentication of the origin of organic matter because of the stability of C-H bonds, and because the lignin’s stable hydrogen and carbon isotopes (δ^2^H_LM_ and δ^13^C_LM_) did not exchange with hydrogen and carbon atoms from source water or organic metabolites^20–24^.

The δ^2^H_LM_ values of plant materials have been shown to record the primary signatures from lignin biosynthesis and the δ^2^H composition (δ^2^H_Pre_) of the local precipitation^21^. This is due to the robust isotope fractionation (ε_app_ = − 216 ± 19 mUr) between plant δ^2^H_LM_ and precipitation δ^2^H_Pre_, which is controlled by air temperature in the mid-latitude regions^25,26^. Most of the studies of δ^2^H_LM_ values have applied them to reconstruct paleoclimate^27–30^, to differentiate biogeochemical processes^20,22^, and to trace the geographical origin of organic matter^31,32^. However, the analytical application of δ^2^H_LM_ to study the degradation of plant materials has been rarely reported. A recent study combined analysis of the organic matter’s methoxy group content with its δ^2^H_LM_ and δ^13^C_LM_ values to investigate the effect of degradation of plant litter on isotopic authenticity^33^. Although the total the total litter mass loss and the change in the composition of the methoxy groups in the litter had no essential impact on the δ^2^H_LM_ signatures during natural biodegradation, we still lack detailed knowledge of the variability in δ^2^H_LM_ during biotic and abiotic degradation, particularly in terms of the different roles and characteristics of bacterial versus fungal degradation. Moreover, previous research focused more on the adsorption capacities and conversion efficiencies of the enzymes for lignin^34^, and we found no study was reported describing the dynamic variations in lignin monomers and their δ^2^H_LM_ characteristics during lignin degradation by different microorganisms. Providing the missing information would be of considerable value in expanding our ability to perform structural characterization of lignin and apply this substance as a biomarker for environmental tracers in biogeochemical cycles and organic matter decomposition.

To address these limitations of our knowledge, we designed the present investigation to compare the variations in lignin during biodegradation of garden biomass. We chose fungal and bacterial strains that had been previously shown to be active in lignin biodegradation, and tested their biodegradation effectiveness both separately and in combination under controlled thermophilic conditions in vitro. The gas chromatograph–mass spectrometry (GC–MS) and gas chromatograph–isotope-ratio mass spectrometry (GC-IRMS) analyses of the degradation residues at 2-day intervals during a 15-day cultivation were performed. We hypothesized that the lignin monomer contents and the δ^2^H_LM_ values would change independently, and would reveal the different relative roles of bacteria versus fungi in the biodegradation. Our specific objectives were to (1) investigate the variability in lignin degradation by *Pseudomonas mandelii*, *Aspergillus fumigatus*, and their co-culture, and (2) evaluate the dynamic characteristics of lignin degradation based on the ratio of the two main monomers and their δ^2^H_LM_ values in the degradation residues. We found that the specialization for lignin methoxy structure and selective hydrolysis during microbial action produced an unbalanced ratio of the two monomers, but that the δ^2^H_LM_ values, with robust fractionation, remained essentially constant.

## Materials and methods

### Materials and microorganisms

We used multisource garden biomass composed of leaf litter, dead wood, and horticultural pruning residues that were collected from the Xi’an Botanical Garden (Shaanxi, China). Each component was cut with pruning shears into fragments approximately 1 cm long and thoroughly mixed to create pooled samples with equal weight proportions of each material. The homogeneous mixtures were then added to conical glass flasks. The main chemical composition of the bulked materials is cellulose (32%), hemicellulose (17%), and total lignin (27%), which contains lignin monomers in the following proportions: G-lignin (10.0%), S-lignin (13.6%), and total methoxy groups (5.7%).

The microbial inoculates used in this study were the bacterium *Pseudomonas mandelii* (strain QL-1, hereafter “PM”), the fungus *Aspergillus fumigatus* (strain QL-4, hereafter “AF”), and the combination of the two strains (combination of QL-1 and QL-4, hereafter “PM + AF”). These lignin-degrading strains were isolated from soil humus in the understorey of a natural forest in the Qinling Mountains in central China in September 2019 and were identified by means of 16S ribosomal DNA and internally transcribed spacer sequencing at Beijing Liuhe BGI Technology Co., Ltd. (Beijing, China) (Supplementary Fig. S1). We used a chromatographic-grade internal standard (tetracosane, 99.5%) and the reaction reagent (hydriodic acid, 55–58%) purchased from Macklin (Shanghai, China). All the other chemicals were of analytical grade and purchased from Kemiou Chemical Reagent Co., Ltd. (Tianjin, China).

### Biodegradation experiment

Before the biodegradation experiment, all biomass materials were thoroughly homogenized using a vortex oscillator (5 min), sterilized at high temperature (121 °C) and pressure (103 kPa) for 20 min, and then dried in an oven at 85 °C for 8 h. To capture the variation in lignin monomers and δ^2^H_LM_ values of the degradation residues, we performed biodegradation experiments both separately for each microbe (PM and AF) and using their combination as a co-culture (PM + AF). For the separate microbial strains, we used liquid suspensions with similar cell concentrations (10^7^ to 10^8^ CFU/mL), which were cultured in Luria–Bertani medium for PM and potato dextrose agar medium for AF, both at 30 °C with shaking 150 rpm. The co-culture suspension was produced by mixing the same total volume (30 mL) used in the single-strain suspensions.

The experimental setup and operation followed the method of Yoon and Ji^35^ with slight modifications. In summary, we added 30 mL of 5 mmol/L phosphoric acid buffer (pH 7.0) to a 250-mL conical flask containing 5 g of the homogenized garden biomass. Subsequently, we inoculated 0.5 mL of the cultured suspension (a 10% volume to substrate mass ratio) into the flask and then sealed the opening with breathable film (Mlbio, Shanghai, China). We used the same volume (0.5 mL) of sterile water as a control treatment, which included 30 mL of the buffer solution. The biodegradation experiments were then performed in the dark using a shaking incubator at 45 °C and 150 rpm for 15 days. We created 84 batches (three replicates in a given measurement period for the three inoculations and the control) for use in seven sampling periods.

### Sample collection and analysis

#### Sample collection

We observed the degradation status daily, and added sterile water, as necessary, to maintain a consistent solution volume. We collected three replicate batches of degradation residues per experiment as analytical samples on days 3, 5, 7, 9, 11, 13, and 15. The initial samples on day 1 were obtained before we began the degradation experiment. The samples were washed three times with 15 mL of sterile water, filtered, and the solids were then dried (85 °C) to constant weight. The degradation weight loss (*DWL*, %) of the garden biomass was calculated as follows:

$$DWL=\left({M}_{\mathrm{b}}-{M}_{\mathrm{a}}\right)/{M}_{\mathrm{b}}\times 100\%,$$

where *M*_b_ is the initial amount of materials (5 g) before biodegradation, and *M*_a_ represents the value after biodegradation for the experimental or control treatment on day *n* of biodegradation. Net degradation loss (*NDL*, %) of the garden biomass was the difference in *DWL* between the inoculation experiment and the uninoculated control. The analytical samples were ground (to a diameter < 0.1 mm) prior to measurement of the lignin monomer content and lignin methoxy δ^2^H_LM_.

#### Lignin monomers composition analysis

Qualitative and quantitative analyses of lignin monomers were performed using the thioacidolysis reaction, which can effectively cleave the ether linkages of lignin monolignols and yield thioester derivatives^36^. The composition of lignin monomers in the degradation residues were measured by using a 7890B gas chromatograph (GC) equipped with G4513A auto-injector, coupled to a 7000D mass spectrometer (GC–MS; Agilent, USA). The GC was fitted with a HP-5MS column (30 m × 0.32 mm × 0.25 µm; Agilent) and the following conditions were employed: inlet temperature 250 °C, injection volume 1 μL, split injection (10:1), initial oven temperature at 150 °C for 2 min, ramp at 20 °C/min to 250 °C and hold for 5 min. Helium was used as the carrier gas at a constant flow of 1.0 mL/min. The following MS conditions were used: electron ionization (EI) mode, ion source temperature 230 °C, interface temperature 250 °C, electron energy 70 eV, solvent delay 3.5 min, scan range from 40 to 650 amu. The GC–MS was run in selective ion monitoring mode for the following molecular ions and retention times: *m/z* 269 for G-lignin derivant at 17.5 min, *m/z* 299 for the S-lignin derivant at 20.8 min, and *m/z* 338 for internal standard tetracosane at 12.7 min.

The final determination of the lignin monomer contents followed the method of Lapierre^37^, where the response factor (*k*) equalled the ratio of the relative concentration to the relative area between the internal standard and the target sample:

$$k=\left({C}_{\mathrm{s}}/{C}_{\mathrm{i}}\right)/\left({A}_{\mathrm{s}}/{A}_{\mathrm{i}}\right),$$

where *k* was equal to 1.5, *C*_s_ and *C*_i_ represent the concentrations of the lignin monomer derivatives and the tetracosane, respectively, and *A*_s_ and *A*_i_ represent the corresponding peak areas. Moreover, we assumed that the thioacidolysis reaction was sufficient and complete in this study, and defined the conversion of substrate and the recovery of internal standard to be 100%^38,39^. We then converted the sample lignin monomer contents into the amounts of G- and S-lignin using the ratio of the molecular weight between the monolignols and their derivatives (0.67 for G-lignin and 0.70 for S-lignin)^36^. Net degradation loss (*NDL*, %) of G-lignin and S-lignin was the difference in lignin monomer contents between the inoculation experiments and the uninoculated control, and the net degradation loss rate (*NDR*, %·day^−1^) was defined as the net degradation loss of lignin monomers at every degradation stage. For more details of the thioacidolysis reaction, GC–MS conditions, and chromatographic chart, please refer to the Supplementary information (Supplementary Figs. S2, S3a; Supplementary Text S1.1).

#### Measurement of the stable hydrogen isotope ratio of lignin methoxy groups

δ^2^H_LM_ values of analytical powdered samples were measured as the headspace iodomethane (CH_3_I), released by the selective substitution reaction between the methoxy groups of the degradation residues and hydriodic acid (HI). We followed the established methods of Keppler et al.^21^ and Greule et al.^40^ with minor modifications^29^. Specifically, 0.5 mL of HI was added to the degradation residues (10 ± 0.5 mg) in a brown crimp glass vial (1.5 mL; Agilent) containing a tiny magnet. The vial was sealed with aluminum caps containing PTFE-lined butyl rubber septa (11-mm crimp and 0.9-mm thickness) and stirred in an oil bath at 120 °C for 30 min. This conversion temperature was a weighted value based on validation by Keppler et al.^21^ at 110 °C and Greule et al.^40^ at 130 °C. After incubating, the sub-samples were allowed to equilibrate at 22 ± 0.5 °C (in an air-conditioned room) for at least 40 min. Finally, an aliquot (80–100 μL) of the CH_3_I was directly injected into the analytical system by using a manual gas-tight syringe (100 μL; Hamilton, USA).

δ^2^H_LM_ values of CH_3_I were measured using a TRACE 1310 GC coupled with an ISOLINK II Delta V Advantage isotope-ratio mass spectrometer (IRMS) via a thermal conversion reactor (ceramic tube [Al_2_O_3_], length 320 mm, 0.5 mm i.d., reactor temperature 1400 °C) (Thermo Fisher, Germany). The measurements were performed at the laboratory of biogeochemistry, Shaanxi Normal University. The GC was fitted with a TG-5MS column (30 m × 0.25 mm × 0.25 µm; Thermo Fisher), and based on the following parameters: inlet temperature 200 °C, split injection (12:1), initial oven temperature at 40 °C for 3.8 min, ramp at 20 °C/min to 80 °C and holding for 1 min, and then ramp at 40 °C/min to a final temperature of 100 °C and hold for 3 min. Helium was used as the carrier gas at a constant flow of 0.8 mL/min. High purity hydrogen gas (99.999%; Beijing AP Baif Gases Industry Co., China) was used as the monitoring gas. The H_3_^+^ factor ranged from 4.7 to 5.0 ppm/nA during the measurement period.

To improve accuracy, the measurement should follow the principle of identical treatment for the analytical samples and reference materials, and the δ^2^H_LM_ values should be normalised by a two-point calibration^37^. However, due to the lack of available commercial samples with true δ^2^H_LM_ values as reference materials, we could only use two homogenized wood samples from different geographical locations and tree species to examine the stability and parallelism of the GC–IRMS results. These two wood samples have been recently measured by Frank Keppler's lab at Heidelberg University against that laboratory’s standards^41^. Hence, the reported δ^2^H_LM_ data in this study were expressed relative to the monitoring hydrogen gas and calibrated against our in-house standards. Furthermore, milli-Ureys (mUr) were used as the unit of isotope δ-values instead of the V-SMOW (Vienna Standard Mean Ocean Water) scale in per mil (‰)^42^. The standard deviations (*n* = 3 or 7, 1σ) ranged from 0.6 to 2.4 mUr. For more details about the substitution reaction, measurement conditions, correction method, and chromatographic charts, please refer to the Supplementary information (Supplementary Fig. S2, S3b and Supplementary Text S1.2).

### Statistical analysis

The reported data are the mean ± standard deviation of the three replicated experiments. The statistical significance of the measurements was evaluated by one-way analysis of variance (ANOVA, with significance at *P* < 0.05), and when the ANOVA results were significant, we used Tukey’s HSD test to identify pairs of values that differed significantly between the degradation experiments.

## Results and discussion

### Differences in the microbial degradation of lignocellulosic biomass

**Figure 1:**
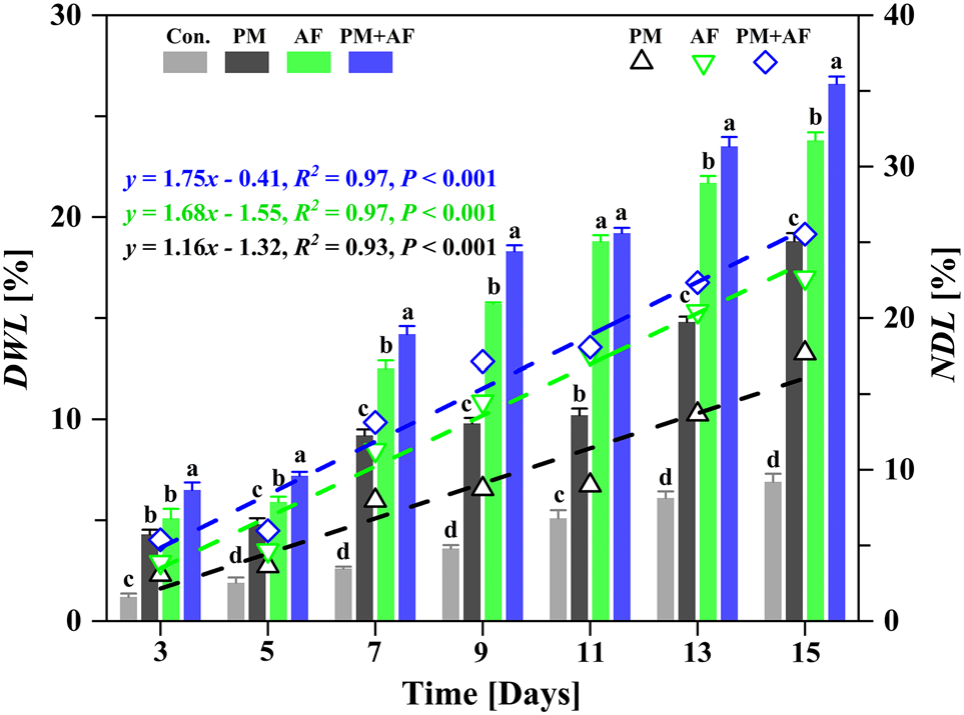
The changes in degradation weight loss (*DWL*) and net degradation loss (*NDL*, the difference in the *DWL* value between the inoculation experiment and the uninoculated control) for garden biomass during the 15-day experiments, with measurements at 2-day intervals. The equations and dashed lines represent the linear fit of the *NDL* over the 15-day experiments. Experiments: Con., uninoculated control; PM, *Pseudomonas mandelii*; AF, *Aspergillus fumigatus*; PM + AF, co-culture of the two microbes. Bars on a given date labelled with different letters differ significantly (*P* < 0.05) between the four treatments.

Figure 1 and Supplementary Table S1 show the degradation weight loss (*DWL*, %) and net degradation loss (*NDL*, %) of garden biomass at 2-day intervals during the study period. At a given degradation stage, both microbes were able to degrade the lignocellulosic biomass, but co-culture of the microorganisms frequently achieved a significantly (*P* < 0.05) greater degradation. In the 15-day degradation experiments, the *DWL* value of the bacterium (PM experiment) increased from 4.3% (day 3) to 18.9% (day 15), and the corresponding *NDL* value increased from 3.1 to 11.9% with a linear fit and a slope of 1.16 (*P* < 0.001). The final *DWL* value for the fungus (AF experiment) increased to 23.9%, and the *NDL* value increased from 3.9 to 17.0% during the study period with a slope of 1.68 (*P* < 0.001). This showed that the fungus could rapidly degrade or ferment the lignocellulose components into various bio-products by means of enzymatic hydrolysis. As expected, the biodegradation efficiency in the co-culture (PM + AF experiment) was generally higher than that of the single strains, with a final *DWL* value as high as 26.7% and a fitted slope of 1.75 (*P* < 0.001) for *NDL*. Usually, a multi-species symbiotic mixture is the best approach to promote diverse enzyme production and synergistic effects^43^. This could be why co-cultivation of different species can trigger more responses to chemical signals than would be possible with a single fungus or bacterium^44^.

The conversion efficiency of the degraded substances showed a large increase starting on day 7, especially in the fungal inoculation experiments (AF and PM + AF). Small molecules such as amino acids are metabolized first by microorganisms, followed by precursors (i.e., cellobiose, xylose, and oligomers) of refractory macromolecular substances^3,45^. Except for some materials with good thermo-solubility, these degraded substances are mainly derived from polysaccharide units and can be preferentially metabolized by microorganisms as carbon and energy sources. Thus, efficient hydrolysis of cellulose and hemicellulose was evident at the early stages of the three inoculation experiments. Previous studies have reported that the lignocellulose-degrading filamentous fungi, including *Aspergillus*, *Trichoderma*, *Penicillium,* and *Chaetomium* species, can efficiently secrete a range of lignocellulosic enzymes, with high extracellular enzymatic activity^2,46^. Although both *A. fumigatus* and *P. mandelii* in this study belong to the group of leaf-litter-decomposing microorganisms, the highest degradation efficiency was obtained in the co-cultivation. According to Miao et al.^47^, the activities of the major cellulases and xylanase secreted by *A. fumigatus* stayed relatively low during the first 3 days, and their peak values with mycelium growth were generally obtained around the 6th day. In contrast, the enzymes from *P. mandelii*, a cold-adapted bacterium, may have displayed low catalytic activity because of the moderate to high temperature (with culturing at 45 °C for 15 days)^48^. Among those temperature-dependent enzymes, extracellular glucosidases and cellobiohydrolases had a substrate preference for the hydrolysis of polysaccharides. Therefore, the combined fungal–bacterial consortium has a unique biodegradation advantage due to synergies among the species and enzymes^49^.

### Degradation dynamics of the lignin monomers

**Figure 2:**
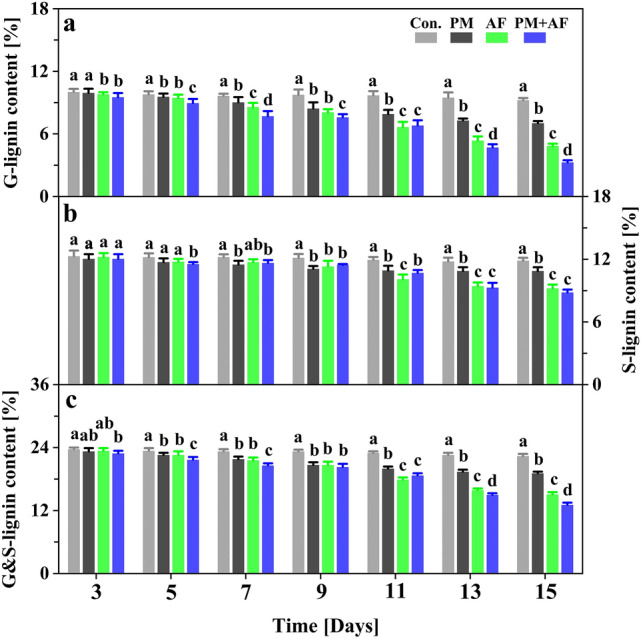
Temporal variations in the content of (**a**) guaiacyl monolignol (G-lignin), (**b**) syringyl monolignol (S-lignin), and (**c**) the sum of the two monolignols (G&S-lignin) in the degradation residues during the 15-day experiments, with measurements at 2-day intervals. Experiments: Con., uninoculated control; PM, *Pseudomonas mandelii*; AF, *Aspergillus fumigatus*; PM + AF, co-culture of the two microbes. Bars on a given date labelled with different letters differ significantly (*P* < 0.05) between the four treatments.

**Figure 3:**
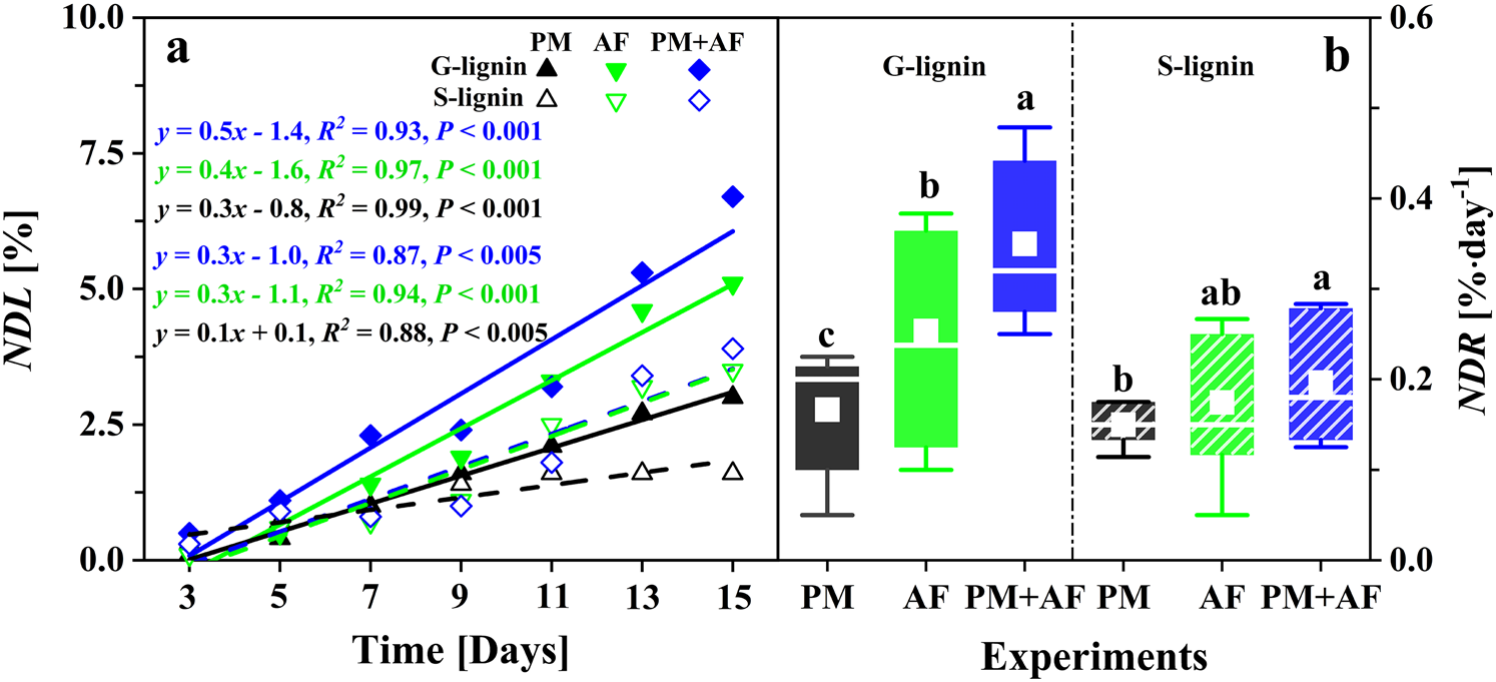
Net loss of guaiacyl monolignol (G-lignin) and syringyl monolignol (S-lignin) monomers in the three inoculation experiments: (**a**) net degradation loss (*NDL*, the difference in lignin monomer contents between the inoculation experiments and the uninoculated control) and (**b**) net degradation loss rate (*NDR*, the *NDL* at the end of the incubation divided by the duration of the incubation, in days). The equations and dashed lines represent the linear fit of the *NDL* versus time over the 15-day experiments. In the boxplots, white squares represent mean values, the horizontal lines represent median values, the boxes represent the 25th to the 75th percentiles, and whiskers represent the 95% confidence interval. Experiments: PM, *Pseudomonas mandelii*; AF, *Aspergillus fumigatus*; PM + AF, co-culture of the two microbes. Bars across all dates labelled with different letters differ significantly (*P* < 0.05) between the four treatments.

The changes in the content of lignin monomers (G- and S-lignin) showed similar patterns in the PM, AF, and PM + AF experiments (Figs. 2, 3). Within the first 5 days, the G-lignin and S-lignin contents decreased slightly, from 10.0% and 13.6% to 9.6% and 13.0%, respectively, for PM, to 9.5% and 13.1% for AF, and to 8.9% and 12.8% for PM + AF (Supplementary Table S2). On subsequent days, the decrease in G-lignin was particularly significant compared to S-lignin (Fig. 2a,b). At the end of the degradation period (day 15), the G-lignin content in the AF and PM + AF fungal experiments had decreased by 5.1 and 6.7 percentage points, respectively, whereas S-lignin decreased by only 3.4 and 3.8 percentage points, respectively (Table 1). Overall, the sum of the G- and S-lignin contents decreased by 4.5, 8.5, and 10.5 percentage points in the three inoculations, respectively (Fig. 2c, Table 1). In addition, the *NDL* values of G-lignin were much larger than those of S-lignin after day 7 (Supplementary Table S3). Although the corresponding linear fits for both lignin monomers were significant over the entire degradation period, the steepness and significance of the equations for the three experiments differed, with the slope greatest for PM + AF, followed by AF and PM (Fig. 3a). This result is consistent with the total degradation weight loss of lignocellulosic biomass, and demonstrates that fast degradation occurred in the co-culture system. Furthermore, the net degradation loss rate (*NDR*) of G-lignin content showed different but significant increases throughout the degradation experiments, whereas for S-lignin the *NDR* increased significantly between PM and PM + AF treatments (Fig. 3b, Supplementary Fig. S4). These results suggest that the fungus had a strong degradation ability and some selectivity for G-lignin, especially in the co-culture mode with both the bacterium and the fungus.

**Table 1:** Variations of the lignin monomer content, methoxy group content, monomer ratio, and δ^2^H_LM_ values in the bulk material and degradation residues from the beginning of biodegradation to the end.

Period (day)	Experiment	*GMC* (%)	*SMC* (%)	*G&SMC* (%)	*MGC* (%)	*G/S* ratio	δ^2^H_LM_ (mUr)
Initial (day 1)	Control	10.0 ± 0.2	13.6 ± 0.3	23.6 ± 0.4	5.7 ± 0.12	0.74 ± 0.01	− 230.3 ± 1.5
Final (day 15)	Control	9.2 ± 0.2a	13.2 ± 0.3a	22.4 ± 0.4a	5.5 ± 0.14a	0.70 ± 0.02a	− 230.4 ± 1.9 a
	PM	7.0 ± 0.2b	12.1 ± 0.3b	19.1 ± 0.3b	4.8 ± 0.12b	0.58 ± 0.00b	− 230.0 ± 2.3 a
	AF	4.9 ± 0.2c	10.2 ± 0.4c	15.1 ± 0.4c	3.9 ± 0.11c	0.47 ± 0.01c	− 227.5 ± 2.2 a
	PM + AF	3.3 ± 0.2d	9.8 ± 0.3c	13.1 ± 0.4d	3.2 ± 0.06d	0.33 ± 0.02d	− 228.1 ± 1.1 a

Experiments: Control, uninoculated control; PM, *Pseudomonas mandelii*; AF, *Aspergillus fumigatus*; PM + AF (P + A), co-culture of the two microbes. *GMC*, guaiacyl (G-lignin) monomer content; *SMC*, syringyl (S-lignin) monomer content; *G&SMC*, G&S-lignin monomer content; *MGC*, methoxy group content; *G/S*, *GMC/SMC*. Values of a parameter labelled with different letters differ significantly (*P* < 0.05).

Previous reports on microbial utilization of lignocellulosic materials and lignin depolymerization demonstrated that lignin was decomposed into small aromatic compounds by extracellular oxidases secreted by multiple microorganisms, including both fungi and bacteria^14,15,50,51^. These ligninolytic enzymes consist of at least two types: heme-containing peroxidases (such as lignin peroxidase, manganese peroxidase, and some versatile peroxidases) and phenol oxidase (laccase). They all can generate unstable free radicals to attack and cleave the chemical bonds. Generally, lignin-degrading fungi can secrete a variety of oxidative enzymes, which have stronger enzymatic hydrolysis selectivity and catalytic efficiency and are therefore better able to break the intra- and inter-monomer linkages and aromatic rings through both specific and nonspecific oxidative pathways^52,53^. In contrast, bacteria decompose lignin slower than fungi, since they have fewer oxidases and fungi are less sensitive to the antibacterial and hydrophobic characteristics of the resulting aromatics^54,55^. As shown in Figs. 2 and 3, the incubation results demonstrated that fungal inoculum has greater ability to decompose lignin, as well as other lignocellulosic substrates. The highest degradation efficiencies for both G-lignin and S-lignin were in the co-culture experiment, which may have resulted from the combined action of different enzymes and synergistic effects among the extracellular enzymes^46,49^.

**Figure 4:**
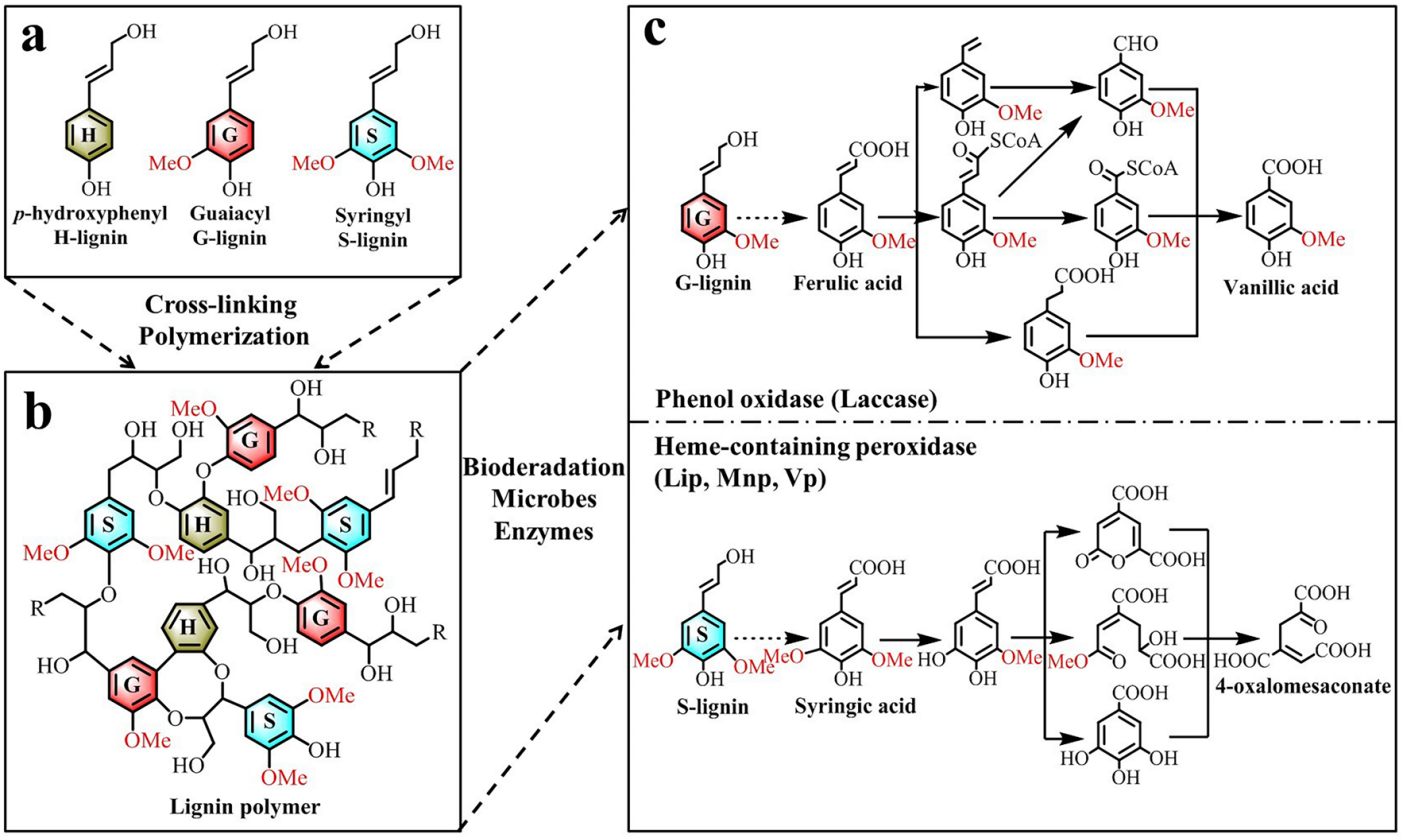
The (**a**) chemical structures, (**b**) polymeric structures, and (**c**) biodegradation pathways for lignin-based aromatic compounds^15^. Lip, lignin peroxidase; Mnp, manganese peroxidase; Vp, versatile peroxidase.

In the biochemical degradation pathways for lignin, lignin polymers are first depolymerized into monomers by microbial catalysis, followed by aromatic catabolism, and are then further degraded by cleavage of aromatic rings and incorporation in the citric acid cycle^15^. The structural difference caused by the methoxy specificity of the different enzymes (Fig. 4a,b) is the main reason why G- and S-lignin follow different aromatic metabolic pathways, with ferulic acid and syringic acid acting as the initial degradation products from the degradation of G-lignin and S-lignin, respectively (Fig. 4c). In general, ferulic acid (from G-lignin) can be transformed into the intermediate vanillic acid via non-oxidative decarboxylation, CoA-dependent β-oxidation/non-β-oxidation, and side chain reduction pathways^56^. Under the catalysis of vanillate-*O*-demethylase, vanillic acid is ultimately demethylated into protocatechuic acid^57^. In contrast, syringic acid (S-lignin) is first demethylated to form the intermediate 3-*O*-methylgallate by tetrahydrofolate-dependent *O*-demethylase, and then catalysed into 4-oxalomesaconate through a series of cleavage, demethylation, and carboxylation pathways via multiple metabolic enzymes^58^. Finally, these low-molecular-weight lignin-derived aromatic substances become sources of carbon and other materials for microbial cell growth and metabolism^54^.

In addition, combined with the irreversibility of methylation in lignin biosynthesis^9^, we observed that S-lignin is more difficult to degrade than G-lignin, possibly due to the chemical stability of the methoxy groups or even to biological inhibition of enzyme activities^59^. For example, GC–MS analysis showed that some monomeric derivatives that contain methoxy groups, such as 3-methylbutanoic acid, 5-methyl-5-propylnonane, 3-methyl-2-butanol, 2-methoxyphenol, and 4-methyltridecane, could be easily detected during the process of lignin degradation^8,60^. The presence of these intermediate metabolites suggests that the release of methoxy groups from aromatic substrates is the major metabolic pathway for lignin depolymerization, although methoxy groups can be used as C1 source for the biosynthesis of methionine^61^. Therefore, our results could indicate that the enzymatic selectivity of microbial metabolism and the structural specificity of methoxy groups together contribute to the observed differences in lignin monomers during biodegradation.

### Variation of the lignin monomer ratios and δ^2^H_LM_ values

**Figure 5:**
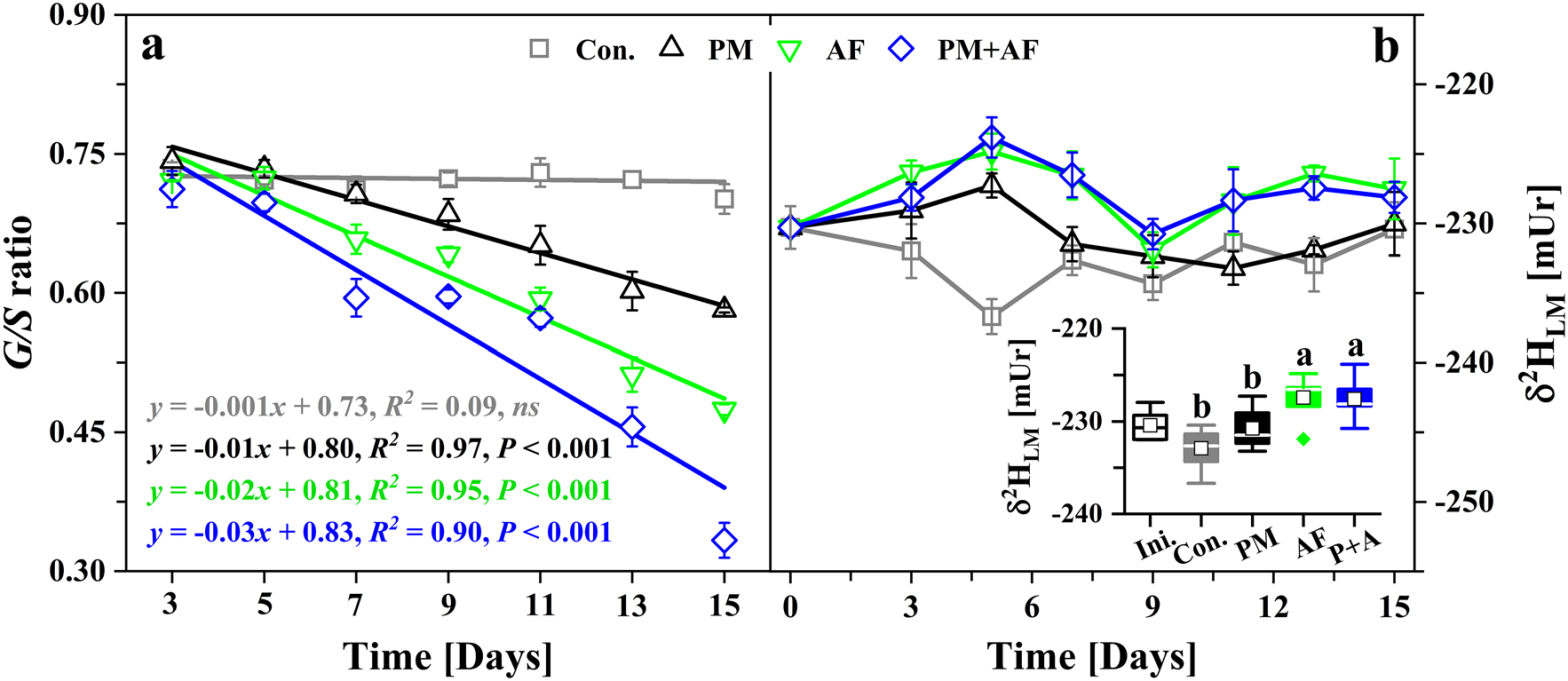
Temporal variations in (**a**) the lignin monomers ratio (G-lignin/S-lignin, *G/S*) and (**b**) the stable hydrogen isotope values (δ^2^H_LM_) of lignin methoxy groups in the degradation residues. The equations and lines represent the linear fit of the *G/S* ratio over the 15-day experiments. In the boxplots, white squares represent mean values, the horizontal lines represent median values, the boxes represent the 25th to the 75th percentiles, whiskers represent the 95% confidence interval, and the green diamond represents outliers. Bars labelled with different letters differ significantly (*P* < 0.05). Ini., initial δ^2^H_LM_ value of the bulk material; Con., uninoculated control; PM, *Pseudomonas mandelii*; AF, *Aspergillus fumigatus*; PM + AF (P + A), co-culture of the two microbes.

We further characterized the changing characteristics of lignin degradation by using the lignin monomer ratios (*G/S*) and the methoxy δ^2^H_LM_ values. The *G/S* ratios of the degradation residues in the three inoculation experiments showed different rates of decrease starting from the initial ratio value of 0.74 (Fig. 5a, Tables 1 and Supplementary Table S4). The largest decrease in the *G/S* ratio occurred in the PM + AF experiment, with a final value of 0.33, followed by the AF experiment (0.47) and the PM experiment (0.58). In contrast, the *G/S* ratio in the uninoculated control experiment remained essentially constant (0.73 ± 0.01) throughout the degradation period (Fig. 5a). Furthermore, based on the theoretical relative molecular mass ratio of methoxy groups to lignin monomers (17.2% for G-lignin and 29.5% for S-lignin), we calculated that the methoxy group content of the degradation residues decreased from the initial 5.7% to a final value of 3.2% (PM + AF), versus 3.9% (AF) and 4.8% (PM) (Table 1). These findings further confirm our observations that G-lignin is more susceptible to degradation than S-lignin and that both forms of degradation occur faster in experiments with fungal inoculation.

The initial mean δ^2^H_LM_ value of the bulk material before the degradation experiments was − 230.4 ± 1.5 mUr, which is close to the value (− 233.6 ± 9.4 mUr, *n* = 119) in our research group’s multi-year measurements of tree-ring wood (manuscript under review). Because these tree wood samples were collected from the Qinling Mountains, just 30 km from where we collected garden biomass samples in the present study, the consistency of the two sets of δ^2^H_LM_ values suggests consistency with the plant source water (i.e., local precipitation). In addition, although the δ^2^H_LM_ values in the different culture experiments fluctuated after around 5 days of degradation, the measured δ^2^H_LM_ values did not change significantly (ANOVA, *P* < 0.05) during the entire degradation period (Supplementary Table S5). Although the experimental material used in this study was a mixture of multi-source garden biomass, plant litter usually contains roughly equal proportions of lignin and pectin^62^. The pectic polysaccharides, another aromatic methoxy donor, are abundant in the cell walls of plant leaves and are easily hydrolysed along with cellulose and hemicellulose^63^. According to Anhäuser et al.^33^, the shift of δ^2^H_LM_ values at beginning of degradation might result from the contribution of pectin, whose methoxy groups have not been analyzed separately from lignin. Furthermore, *S*-adenosylmethionine is the common methoxyl group donor for G-lignin and S-lignin biosynthesis, but the aromatic ring is methylated at the 3 position, the 5 position, or both via different *O*-methyltransferases^9^. Therefore, the presence of these variations in the δ^2^H_LM_ values suggests that the production of methoxy groups from lignin monomers and pectin is not negligible^33^.

Significantly, the co-culture experiment showed the greatest *G/S* ratio decrease and loss of methoxy groups, did not show the strongest δ^2^H_LM_ enrichment or depletion, which exhibited similar variation and similar mean values (the average during the 15-day degradation period) to the values in the fungal inoculation. In contrast, the observed 3.0-mUr decrease of the δ^2^H_LM_ values in the bacterial experiment was obvious. In addition to the fact that methoxy-rich pectin is easier to degrade, probably resulting in a more positive isotopic signature, isotopic fractionation during microbial enzymatic hydrolysis should be considered^64^. Biodegradation can lead to an enrichment of residual substrates, because of the lighter isotopes can be more easily utilized by microbes^65^. According to Fischer et al*.*^66^, isotopic fractionation of hydrogen was evident during aerobic hydroxylation of benzene rings during biodegradation, and some isotope enrichment factors for a specific biodegradation pathway may be caused by a mixture of degradation materials, cultivation conditions, and complex enzymatic reactions. This result was consistent with the demethylation of lignin aromatic compounds that we observed, with greater enrichment of δ^2^H_LM_ values in the experiments with fungal inoculation.

Even though the final (day 15) δ^2^H_LM_ values (which ranged from − 227.5 to − 230.4 mUr) in the four experiments were not significantly different (ANOVA, *P* < 0.05) from the initial value (− 230.3 mUr), we observed slight enrichment of the lignin δ^2^H_LM_ values during degradation (Fig. 5b, Table 1 and Supplementary Table S5). However, some analytical uncertainties^41,67^, such as the substrate homogeneity, the reference standard used, the injection volume, baseline drift, natural variation (i.e., a high standard deviation), and scale compression, were away from the actual mean. In this study, some standard deviation (*n* = 3, with a range from 0.9 to 2.6 mUr) were greater than or equal to 1.5 mUr, which is clearly greater than what would be expected for ideal isotopic measurements^22,25^. Overall, though the loss of pectin from the leaf litter and microbial metabolic isotope fractionation might be responsible for the observed fluctuation of the δ^2^H_LM_ values during the degradation of lignin, it appears that these processes did not did not depend on the decrease of the *G/S* ratio or the loss of methoxy groups. However, before we can apply lignin-specific δ^2^H_LM_ values to evaluate the degradation dynamics of lignocellulosic materials or as a proxy for lignin deposition from plant litter into soil humus, we require more detailed information about the differences in δ^2^H_LM_ values between G- and S-lignin.

## Conclusion

Our analyses confirmed our hypothesis that the lignin monomers and their δ^2^H_LM_ values would change independently and reveal different relative roles of bacteria and fungi in biodegradation. The lignin-depolymerizing ability of *Aspergillus fumigatus*, alone or in co-culture, was stronger than that of *Pseudomonas mandelii* alone. G-lignin was more easily degraded than S-lignin in all experiments. The measured δ^2^H_LM_ values of the lignin methoxy groups in degradation residues showed no significant dependence on the lignin monomer content, *G/S* ratio, or loss of methoxy groups. This indicated that the δ^2^H_LM_ signal in biodegradation of lignocellulosic material or lignin mineralization can be used as a biomarker for the lignin input from plant litter to organic soil. However, it is not clear whether the δ^2^H_LM_ values vary with respect to the proportions of plant lignin monomers. The differences in δ^2^H_LM_ values between G- and S-lignin should be further investigated by separating the two monomers or determining their isotopic values separately, thereby providing additional insights into lignin biodegradation.

## Supplementary Information

Supplementary Information.

## Data Availability

The datasets used and/or analysed during the current study are available from the corresponding author on reasonable request.

## Abbreviations

Al_2_O_3_: Aluminium oxide
AF: *Aspergillus fumigatus* Strain
CoA: Coenzyme A
CFU: Colony-forming unit
CH_3_I: Iodomethane
Control: Uninoculated control experiment
δ^2^H_LM_: Stable hydrogen isotope ratio in lignin methoxy groups
δ^2^H_Pre_: Stable hydrogen isotope ratio in precipitation
*DWL*: Degradation weight loss
G-lignin: Guaiacyl monolignol
GC: Gas chromatograph
HI: Hydriodic acid
H-lignin: *p*-Hydroxyphenyl monolignol
IRMS: Isotope-ratio mass spectrometer
MS: Mass spectrometer
*NDL*: Net degradation loss
*NDR*: Net degradation loss rate
OCH_3_: Methoxy group
PM: *Pseudomonas mandelii* Strain
PM + AF: The co-culture of *Pseudomonas mandelii* and *Aspergillus fumigatus*
PTFE: Polytetrafluoroethylene
S-lignin: Syringyl monolignol

## References

[1] Liczbiński P, Borowski S (2021). Effect of hyperthermophilic pretreatment on methane and hydrogen production from garden waste under mesophilic and thermophilic conditions. Bioresour. Technol..

[2] Kundariya N, Mohanty SS, Varjani S, Ngo HH, Wong JWC, Taherzadeh MJ, Chang JS, Ng HY, Kim SH, Bui XT (2021). A review on integrated approaches for municipal solid waste for environmental and economical relevance: Monitoring tools, technologies, and strategic innovations. Bioresour. Technol..

[3] Onwosi CO, Igbokwe VC, Odimba JN, Eke IE, Nwankwoala MO, Iroh IN, Ezeogu LI (2017). Composting technology in waste stabilization: On the methods, challenges and future prospects. J. Environ. Manag..

[4] Wainaina W, Awasthi MK, Sarsaiya MK, Chen H, Singh E, Kumar A, Ravindran B, Awasthi SK, Liu T, Duan YM, Kumar S, Zhang ZQ, Taherzadeh MJ (2020). Resource recovery and circular economy from organic solid waste using aerobic and anaerobic digestion technologies. Bioresour. Technol..

[5] Awasthi MK, Sarsaiya S, Wainaina S, Rajendrdan K, Wang Q, Kumar S, Duan Y, Awasthi SK, Pandey A, Chen H, Taherzadeh M (2019). A critical review of organic manure biorefinery models toward sustainable circular bioeconomy: Technological challenges, advancements, innovations, and future perspectives. Renew. Sustain. Energy Rev..

[6] Cheng HH, Whang LM (2022). Resource recovery from lignocellulosic wastes via biological technologies: Advancements and prospects. Bioresour. Technol..

[7] Lambertz C, Ece S, Fischer R, Commandeur U (2016). Progress and obstacles in the production and application of recombinant lignin-degrading peroxidases. Bioengineered.

[8] Riyadi FA, Tahir AA, Yusof N, Sabri NSA, Noor MJMM, Akhir FN, Othman N, Zakaria Z, Hara H (2020). Enzymatic and genetic characterization of lignin depolymerization by *Streptomyces* sp. S6 isolated from a tropical environment. Sci. Rep..

[9] Vanholme R, Meester BD, Ralph J, Boerjan W (2019). Lignin biosynthesis and its integration into metabolism. Curr. Opin. Biotechnol..

[10] Asina F, Brzonova I, Voeller K, Kozliak E, Kubatova A, Yao B, Ji Y (2016). Biodegradation of lignin by fungi, bacteria and laccases. Bioresour. Technol..

[11] Patil V, Adhikari S, Cross P, Jahromi H (2020). Progress in the solvent depolymerization of lignin. Renew. Sustain. Energy Rev..

[12] Chio C, Sain M, Qin W (2019). Lignin utilization: A review of lignin depolymerization from various aspects. Renew. Sustain. Energy Rev..

[13] de Gonzalo G, Colpa DI, Habib MHM, Fraaije MW (2016). Bacterial enzymes involved in lignin degradation. J. Biotechnol..

[14] Kamimura N, Sakamoto S, Mitsuda N, Masai E, Kajita S (2019). Advances in microbial lignin degradation and its applications. Curr. Opin. Biotechnol..

[15] Weng CH, Peng XW, Han YJ (2021). Depolymerization and conversion of lignin to value-added bioproducts by microbial and enzymatic catalysis. Biotechnol. Biofuels.

[16] Falade AO, Nwodo UU, Iweriebor BC, Green E, Mabinya LV, Okoh AI (2017). Lignin peroxidase functionalities and prospective applications. Microbiology.

[17] Jia LL, Qin YJ, Wang J, Zhang JH (2020). Lignin extracted by γ-valerolactone/water from corn stover improves cellulose enzymatic hydrolysis. Bioresour. Technol..

[18] Morya R, Kumar M, Thakur IS (2021). Bioconversion of syringyl lignin into malic acid by *Burkholderia* sp. ISTR5. Bioresour. Technol..

[19] McRoberts WC, Keppler F, Harper DB, Hamilton JTG (2015). Seasonal changes in chlorine and methoxyl content of leaves of deciduous trees and their impact on release of chloromethane and methanol at elevated temperatures. Environ. Chem..

[20] Keppler F, Kalin RM, Harper DB, McRoberts WC, Hamilton JTG (2004). Carbon isotope anomaly in the major plant C1 pool and its global biogeochemical implications. Biogeosciences.

[21] Keppler F, Harper DB, Kalin RM, Meier-Augenstein W, Farmer N, Davis S, Schmidt HL, Brown DM, Hamilton JTG (2007). Stable hydrogen isotope ratios of lignin methoxyl groups as a paleoclimate proxy and constraint of the geographical origin of wood. New Phytol..

[22] Keppler F, Hamilton JTG (2008). Tracing the geographical origin of early potato tubers using stable hydrogen isotope ratios of methoxyl groups. Isot. Environ. Health Stud..

[23] Keppler F, Harper DB, Greule M, Ott U, Sattler T, Schöler HF, Hamilton JTG (2014). Chloromethane release from carbonaceous meteorite affords new insight into Mars lander findings. Sci. Rep..

[24] Greule M, Rossmann A, Schmidt HL, Mosandl A, Keppler F (2015). A stable isotope approach to assessing water loss in fruits and vegetables during storage. J. Agric. Food Chem..

[25] Greule M, Wieland A, Keppler F (2021). Measurements and applications of δ^2^H values of wood lignin methoxy groups for paleoclimatic studies. Quat. Sci. Rev..

[26] Porter TJ, Anhäuser T, Halfar J, Keppler F, Csank AZ, Williams CJ (2022). Canadian arctic Neogene temperatures reconstructed from hydrogen isotopes of lignin-methoxy groups from sub-fossil wood. Paleoceanogr. Paleoclimatol..

[27] Feakins SJ, Ellsworth PV, Sternberg LDSL (2013). Lignin methoxyl hydrogen isotope ratios in a coastal ecosystem. Geochim. Cosmochim. Acta.

[28] Anhäuser T, Hook B, Halfar J, Greule M, Keppler F (2018). Earliest Eocene cold period and polar amplification—insights from δ^2^H values of lignin methoxyl groups of mummified wood. Palaeogeogr. Palaeocl..

[29] Lu QQ, Liu XH, Anhäuser T, Keppler F, Wang YB, Zeng XM, Zhang QL, Zhang LN, Wang KY, Zhang Y (2020). Tree-ring lignin proxies in *Larix gmelinii* forest growing in a permafrost area of northeastern China: Temporal variation and potential for climate reconstructions. Ecol. Indic..

[30] Wang YB, Liu XH, Anhäuser T, Lu QQ, Zeng XM, Zhang QL, Wang KY, Zhang LN, Zhang Y, Keppler F (2020). Temperature signal recorded in δ^2^H and δ^13^C values of wood lignin methoxyl groups from a permafrost forest in northeastern China. Sci. Total Environ..

[31] Greule M, Tumino LD, Kronewald T, Hener U, Schleucher J, Mosandl A, Keppler F (2010). Improved rapid authentication of vanillin using δ^13^C and δ^2^H values. Eur. Food Res. Technol..

[32] van Leeuwen KA, Prenzler PD, Ryan D, Paolini M, Camin F (2018). Differentiation of wood-derived vanillin from synthetic vanillin in distillates using gas chromatography/combustion/isotope ratio mass spectrometry for δ^13^C analysis. Rapid Commun. Mass Spectrom..

[33] Anhäuser T, Greule M, Zech M, Kalbitz K, McRoberts C, Keppler F (2015). Stable hydrogen and carbon isotope ratios of methoxyl groups during plant litter degradation. Isot. Environ. Health S..

[34] Chauhan PS (2020). Role of various bacterial enzymes in complete depolymerization of lignin: a review. Biocatal. Agric. Biotechnol..

[35] Yoon CS, Ji DS (2005). Effects of in vitro degradation on the weight loss and tensile properties of PLA/LPCL/HPCL blend fibers. Fiber. Polym..

[36] Harman-Ware AE, Foster C, Happs RM, Doeppke C, Meunier K, Gehan J, Yue FX, Lu FC, Davis M (2016). Quantitative analysis of lignin monomers by a thioacidolysis method tailored for higher-throughput analysis. J. Biotechnol..

[37] Lapierre C, Monties B, Rolando C (1986). Thioacidolysis of poplar lignins: identification of monomeric syringyl products and characterization of guaiacyl–syringyl lignin fractions. Holzforschung.

[38] Robinson AR, Mansfield SD (2009). Rapid analysis of poplar lignin monomer composition by a streamlined thioacidolysis procedure and near-infrared reflectance-based prediction modeling. Plant J..

[39] Wu L, Rencoret J, Lu FC, Karlen SD, Smith BG, Harris PJ, del Río JC, Ralph J (2016). Tricin-lignins: Occurrence and quantitation of tricin in relation to phylogeny. Plant J..

[40] Greule M, Rossmann A, Hamilton JTG, Keppler F (2008). A rapid and precise method for determination of D/H ratios of plant methoxyl groups. Rapid Commun. Mass Sp..

[41] Greule M, Moossen H, Lloyd MK, Geilmann H, Brand WA, Eiler JM, Qi HP, Keppler F (2020). Three wood isotopic reference materials for δ^2^H and δ^13^C measurements of plant methoxy groups. Chem. Geol..

[42] Brand WA, Coplen TB (2012). Stable isotope deltas: Tiny, yet robust signatures in nature. Isot. Environ. Health. Stud..

[43] Cui TW, Yuan B, Guo HW, Tian H, Wang WM, Ma YQ, Li CZ, Fei Q (2021). Enhanced lignin biodegradation by consortium of white rot fungi: Microbial synergistic effects and product mapping. Biotechnol. Biofuels..

[44] Vipotnik Z, Michelin M, Tavares T (2021). Ligninolytic enzymes production during polycyclic aromatic hydrocarbons degradation: Effect of soil pH, soil amendments and fungal co-cultivation. Biodegradation.

[45] Guo XX, Liu HT, Wu SB (2019). Humic substances developed during organic waste composting: Formation mechanisms, structural properties, and agronomic functions. Sci. Total Environ..

[46] Marmann A, Aly A, Lin WH, Wang BG, Proksch P (2014). Co-cultivation—A powerful emerging tool for enhancing the chemical diversity of microorganisms. Mar. Drugs.

[47] Miao JX, Wang MM, Ma L, Li T, Huang QW, Liu DY, Shen QR (2019). Effects of amino acids on the lignocellulose degradation by *Aspergillus fumigatus* Z5: Insights into performance, transcriptional, and proteomic profiles. Biotechnol. Biofuels.

[48] Lee CW, Kim JY, Hong SH, Goo BL, Lee SY, Jang SH (2013). Cloning, expression, and characterization of a recombinant esterase from cold-adapted *Pseudomonas mandelii*. Appl. Biochem. Biotechnol..

[49] Meehnian H, Jana AK, Jana MM (2017). Pretreatment of cotton stalks by synergistic interaction of *Daedalea flavida* and *Phlebia radiata* in co-culture for improvement in delignification and saccharification. Int. Biodeterior. Biodegrad..

[50] Zhang ST, Xiao JL, Wang G, Chen G (2021). Enzymatic hydrolysis of lignin by ligninolytic enzymes and analysis of the hydrolyzed lignin products. Bioresour. Technol..

[51] Atiwesh G, Parrish CC, Banoub J, Le TT (2022). Lignin degradation by microorganisms: A review. Biotechnol. Progr..

[52] Chi Y, Hatakka A, Maijala P (2007). Can co-culturing of two white-rot fungi increase lignin degradation and the production of lignin-degrading enzymes?. Int. Biodeterior. Biodegrad..

[53] Andlar M, Rezic T, Mardetko N, Kracher D, Ludwig R, Santek B (2018). Lignocellulose degradation: An overview of fungi and fungal enzymes involved in lignocellulose degradation. Eng. Life Sci..

[54] Bugg TD, Ahmad M, Hardiman EM, Singh R (2011). The emerging role for bacteria in lignin degradation and bio-product formation. Curr. Opin. Biotechnol..

[55] Kumar M, You S, Beiyuan J, Luo G, Gupta J, Kumar S, Singh L, Zhang S, Tsang DCW (2021). Lignin valorization by bacterial genus *Pseudomonas*: State-of-the-art review and prospects. Bioresour. Technol..

[56] Graf N (2014). Genetic engineering of *Pseudomonas putida* KT2440 for rapid and high-yield production of vanillin from ferulic acid. Appl. Microbiol. Biotechnol..

[57] Plaggenborg R, Overhage J, Loos A, Archer JA, Lessard P, Sinskey AJ, Steinbüchel A, Priefert H (2006). Potential of *Rhodococcus* strains for biotechnological vanillin production from ferulic acid and eugenol. Appl. Microbiol. Biotechnol..

[58] Kasai D, Masai E, Miyauchi K, Katayama Y, Fukuda M (2005). Characterization of the gallate dioxygenase gene: Three distinct ring cleavage dioxygenases are involved in syringate degradation by *Sphingomonas paucimobilis* SYK-6. J. Bacteriol..

[59] Venkatesagowda B, Dekker RFH (2021). Microbial demethylation of lignin: Evidence of enzymes participating in the removal of methyl/methoxyl groups. Enzyme. Microb. Technol..

[60] Mathews SL, Grunden AM, Pawlak J (2016). Degradation of lignocellulose and lignin by *Paenibacillus glucanolyticus*. Internat. Biodeterior. Biodegrad..

[61] Peng Y, Nicastro KH, Epps TH, Wu C (2021). Methoxy groups reduced the estrogenic activity of lignin-derivable replacements relative to bisphenol A and bisphenol F as studied through two in vitro assays. Food Chem..

[62] Caffall KH, Mohnen D (2009). The structure, function, and biosynthesis of plant cell wall pectic polysaccharides. Carbohydr. Res..

[63] Das S, Majumdar B, Saha AR (2015). Biodegradation of plant pectin and hemicelluloses with three novel *Bacillus pumilus* strains and their combined application for quality jute fibre production. Agric. Res..

[64] Elsner M (2010). Stable isotope fractionation to investigate natural transformation mechanisms of organic contaminants: Principles, prospects and limitations. J. Environ. Monit..

[65] Cui MC, Zhang WB, Fang J, Liang QQ, Liu DX (2017). Carbon and hydrogen isotope fractionation during anaerobic biodegradation of quinoline and 3-methylquinolin. Appl. Microbiol. Biotechnol..

[66] Fischer A, Gehre M, Breitfeld J, Richnow HH, Vogt C (2009). Carbon and hydrogen isotope fractionation of benzene during biodegradation under sulfate-reducing conditions: A laboratory to field site approach. Rapid Commun. Mass Spectrom..

[67] Lee HJ, Feng XJ, Mastalerz M, Feakins SJ (2019). Characterizing lignin: Combining lignin phenol, methoxy quantification, and dual stable carbon and hydrogen isotopic techniques. Org. Geochem..

