# Supplementary material for: Variations in lignin monomer contents and stable hydrogen isotope ratios in methoxy groups during the biodegradation of garden biomass
Source: Sci Rep. 2022 May 24;12:8734. doi: 10.1038/s41598-022-12689-1 (PMC9130509; doi:10.1038/s41598-022-12689-1)
Supplement: Supplementary file 1 — Supplementary Information. [file 41598_2022_12689_MOESM1_ESM.pdf]

Figures, tables, and measurements

Figure S1. Identification and phylogenetic tree for the microbial strains used in the biodegradation experiments. Strains (a) QL-1 and (b) QL-4 were isolated from the humus-rich soil under a natural forest in the Qinling Mountains, and were identified as subspecies of *Pseudomonas mandelii* HQ202858.1 (hereafter PM) and *Aspergillus fumigatus* MG827163.1 (hereafter AF), respectively.

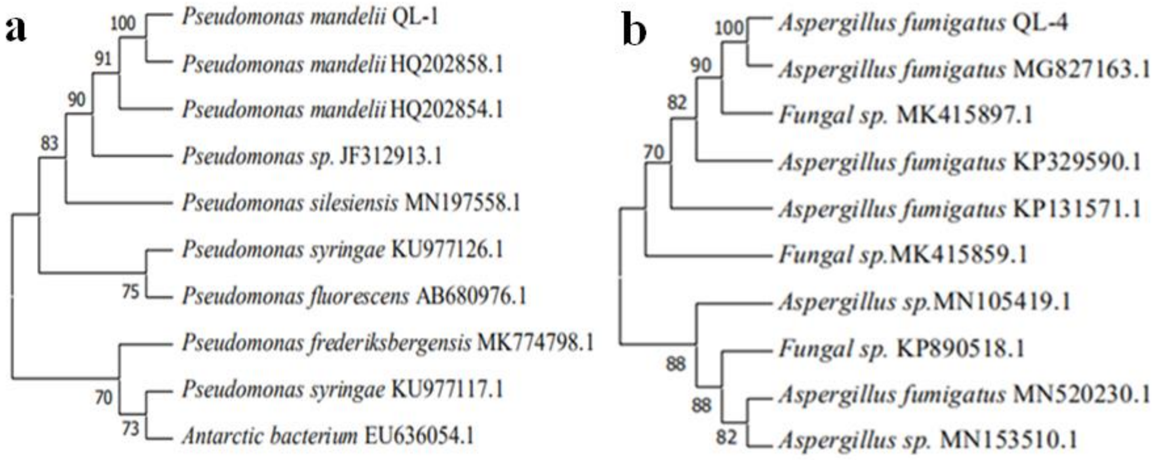

Figure S2. Lignin polymers and their substitution reactions. Lignin in garden biomass is an irregular biopolymer, generally constructed from phenylpropane units, with abundant guaiacyl (G) and syringyl (S) monomers, a small amount of *p*-hydroxyphenyl lignin (H), and other chain-starting monomers via (a) *β*-O-dehydrogenated polymerization, which requires synergistic participation by several enzymes. We analyzed (b) the lignin monomer (G and S) contents, and (c) the stable hydrogen isotope ratio (δ^2H_{LM}) of the lignin methoxy (MeO) groups through a series of substitution derivation reactions at specific sites.

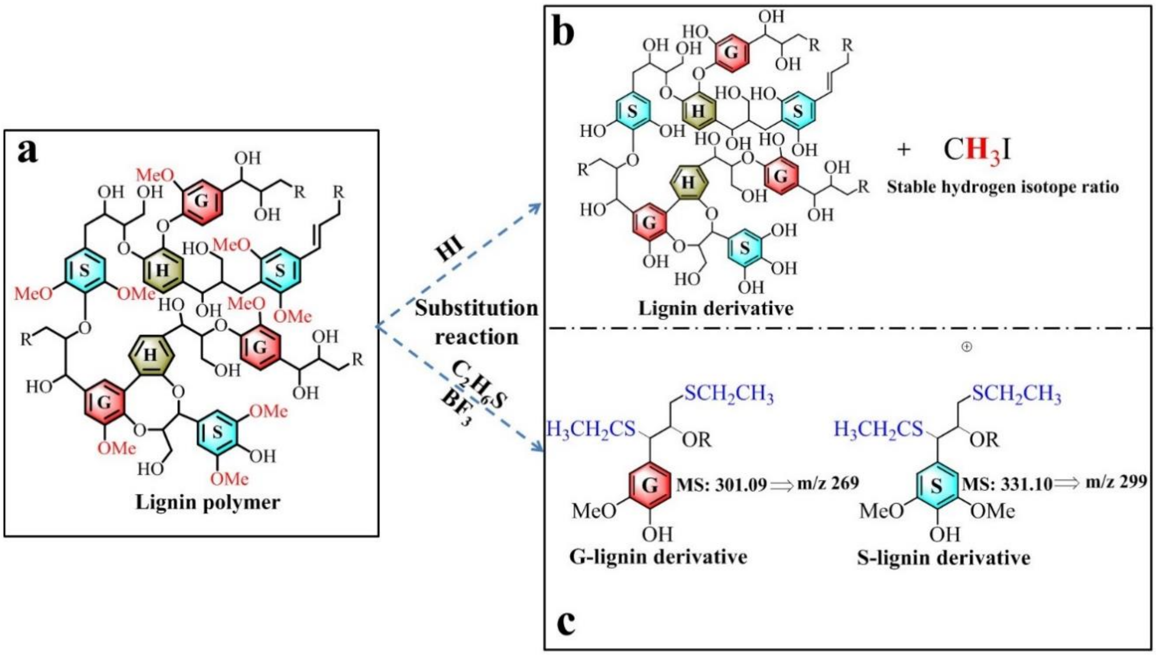

Figure S3. The chromatograms of lignin monomer determination in the degradation residues. The chromatographic charts for (a) the lignin monomer contents and (b) the methoxy group δ^2H_{LM} values.

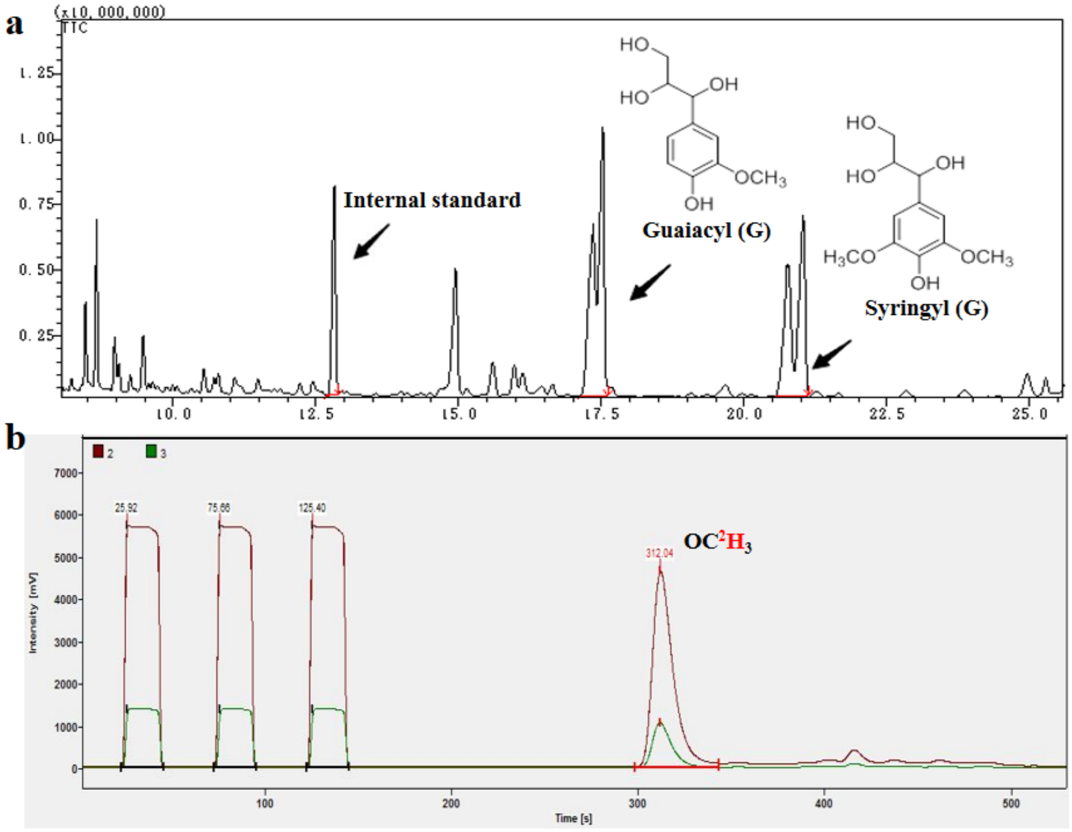

Figure S4. Temporal variations in net degradation loss rate (*NDR*, net degradation loss every degradation stage) of G-lignin and S-lignin content during the 15-day experiments at 2-day intervals. Italics lines and equations represent the strength of the linear fit of the *NDR* over the 15-day experiments. Experiments: PM, *Pseudomonas mandelii*; AF, *Aspergillus fumigatus*; PM+AF, co-culture of the two microbes.

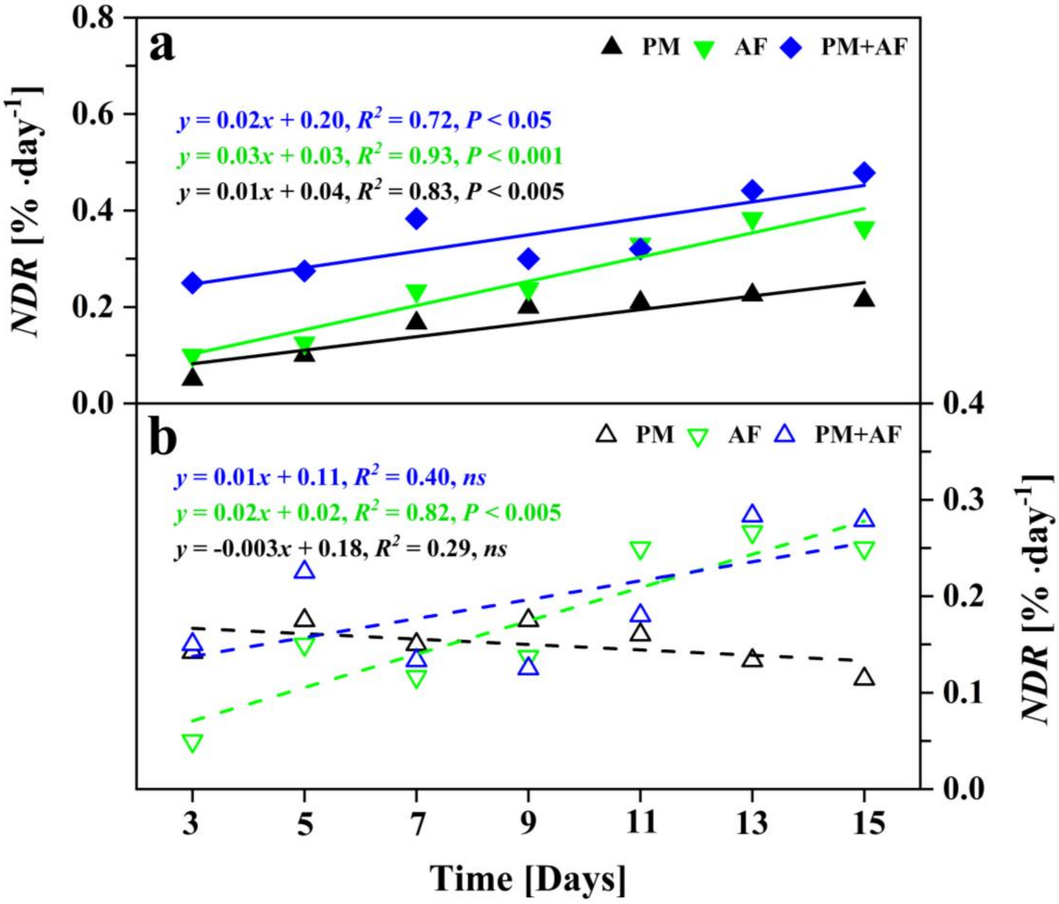

**Experiment**	**Degradation weight loss (*DWL*) of garden biomass (%)**						
	**3 days**	**5 days**	**7 days**	**9 days**	**11 days**	**13 days**	**15 days**
**Control**	1.2 ± 0.2 cE	1.9 ± 0.3 dDE	2.6 ± 0.1 dD	3.6 ± 0.2 dC	5.1 ± 0.4 cB	6.1 ± 0.3 dA	6.9 ± 0.4 dA
**PM**	4.3 ± 0.2 bE	4.8 ± 0.3 cE	9.2 ± 0.3 cD	9.9 ± 0.3 cCD	10.2 ± 0.3 bC	14.9 ± 0.3 cB	18.8 ± 0.4 cA
**AF**	5.1 ± 0.5 bF	5.9 ± 0.3 bF	12.5 ± 0.4 bE	15.7 ± 0.1 bD	18.8 ± 0.3 aC	21.6 ± 0.3 bB	23.9 ± 0.4 bA
**PM+AF**	6.6 ± 0.4 aE	7.2 ± 0.4 aE	14.3 ± 0.2 aD	18.3 ± 0.5 aC	19.3 ± 0.3 aC	23.5 ± 0.2 aB	26.7 ± 0.4 aA

Table S1. Degradation weight loss (*DWL*) of garden biomass during the 15-day experiments at 2-day intervals. Experiments: Control, uninoculated control; PM, *Pseudomonas mandelii*; AF, *Aspergillus fumigatus*; PM+AF, co-culture of the two microbes. Different lower- and upper-case letters represent a significant difference ($P < 0.05$) at different experiments and different stages.

Experiment	Lignin monomer	Lignin monomer content of the degradation residues (%)						
		3 days	5 days	7 days	9 days	11 days	13 days	15 days
**Control**	**G-lignin**	10.0 ± 0.3 aA	9.8 ± 0.3 aAB	9.6 ± 0.2 aB	9.8 ± 0.5 aAB	9.7 ± 0.4 aAB	9.5 ± 0.5 aBC	9.2 ± 0.2 aC
	**S-lignin**	13.7 ± 0.4 aA	13.6 ± 0.4 aA	13.6 ± 0.3 aA	13.5 ± 0.4 aA	13.3 ± 0.3 aA	13.1 ± 0.4 aA	13.2 ± 0.3 aA
	**G&S-lignin**	23.7 ± 0.3 aA	23.3 ± 0.5 aAB	23.2 ± 0.4 aAB	23.2 ± 0.3 aAB	23.0 ± 0.3 aAB	22.6 ± 0.4 aB	22.4 ± 0.4 aB
**PM**	**G-lignin**	9.9 ± 0.4 aA	9.6 ± 0.3 bAB	9.0 ± 0.5 bB	8.4 ± 0.6 bBC	7.9 ± 0.4 bC	7.3 ± 0.2 bD	7.0 ± 0.2 bD
	**S-lignin**	13.4 ± 0.5 aA	13.0 ± 0.4 aA	12.8 ± 0.4 bAB	12.3 ± 0.3 bB	12.1 ± 0.5 bB	12.1 ± 0.4 bB	12.1 ± 0.4 bB
	**G&S-lignin**	23.3 ± 0.6 abA	22.6 ± 0.4 bAB	21.8 ± 0.5 bB	20.7 ± 0.5 bC	20.1 ± 0.4 bCD	19.4 ± 0.4 bD	19.1 ± 0.3 bDA
**AF**	**G-lignin**	9.8 ± 0.2 bA	9.5 ± 0.3 bA	8.6 ± 0.4 cB	8.1 ± 0.3 bC	6.7 ± 0.5 cD	5.4 ± 0.4 cE	4.9 ± 0.2 cF
	**S-lignin**	13.6 ± 0.4 aA	13.1 ± 0.3 aAB	13.0 ± 0.3 abAB	12.6 ± 0.6 bB	11.2 ± 0.5 cC	10.5 ± 0.3 cD	10.2 ± 0.4 cD
	**G&S-lignin**	23.4 ± 0.5 abA	22.5 ± 0.7 bB	21.6 ± 0.5 bC	20.6 ± 0.6 bD	17.9 ± 0.4 cE	15.9 ± 0.3 cF	15.1 ± 0.4 cF
**PM+AF**	**G-lignin**	9.5 ± 0.4 bA	8.9 ± 0.4 cB	7.7 ± 0.5 dC	7.6 ± 0.3 cC	6.8 ± 0.5 cD	4.7 ± 0.3 dE	3.3 ± 0.2 dF
	**S-lignin**	13.4 ± 0.5 aA	12.8 ± 0.2 bA	12.9 ± 0.3 bA	12.7 ± 0.1 bA	11.9 ± 0.3 bB	10.3 ± 0.5 cC	9.8 ± 0.3 cC
	**G&S-lignin**	22.9 ± 0.5 bA	21.8 ± 0.5 cB	20.6 ± 0.4 cC	20.3 ± 0.6 bC	18.7 ± 0.4 cD	15.0 ± 0.3 dE	13.1 ± 0.4 dF

Table S2. Temporal variations in lignin monomer (G-lignin, S-lignin, and the sum of two forms) content of the degradation residues during the 15-day experiments at 2-day intervals. Experiments: Control, uninoculated control; PM, *Pseudomonas mandelii*; AF, *Aspergillus fumigatus*; PM+AF, co-culture of the two microbes. Different lower- and upper-case letters represent a significant difference (P < 0.05) at different experiments and different stages.

Experiment	Lignin monomer	Net degradation loss (*NDL*) of lignin monomers content in the three inoculation experiments (%)						
		3 days	5 days	7 days	9 days	11 days	13 days	15 days
**PM**	**G-lignin**	0.1	0.4	1.0	1.6	2.1	2.7	3.0
	**S-lignin**	0.3	0.7	0.9	1.4	1.6	1.6	1.6
**AF**	**G-lignin**	0.2	0.5	1.4	1.9	3.3	4.6	5.1
	**S-lignin**	0.1	0.6	0.7	1.1	2.5	3.2	3.5
**PM+AF**	**G-lignin**	0.5	1.1	2.3	2.4	3.2	5.3	6.7
	**S-lignin**	0.3	0.9	0.8	1.0	1.8	3.4	3.9

Table S3. Temporal variations in net degradation loss (*NDL*, difference in lignin monomer contents between the inoculation experiments and the uninoculation control) of lignin monomers during the 15-day experiments at 2-day intervals. Experiments: PM, *Pseudomonas mandelii*; AF, *Aspergillus fumigatus*; PM+AF, co-culture of the two microbes.

Experiment	Lignin monomer ratio (G/S) between G-lignin and S-lignin in the degradation residues						
	3 days	5 days	7 days	9 days	11 days	13 days	15 days
**Control**	0.73 ± 0.01 aA	0.72 ± 0.00 aA	0.71 ± 0.01 aA	0.72 ± 0.01 aA	0.73 ± 0.02 aA	0.72 ± 0.01 aA	0.70 ± 0.02 aA
**PM**	0.74 ± 0.02 aA	0.73 ± 0.01 aA	0.71 ± 0.01 abA	0.69 ± 0.02 aAB	0.65 ± 0.02 bB	0.60 ± 0.02 bBC	0.58 ± 0.00 bC
**AF**	0.72 ± 0.00 aA	0.72 ± 0.01 aA	0.66 ± 0.02 bB	0.64 ± 0.01 bB	0.59 ± 0.01 cC	0.51 ± 0.02 cD	0.47 ± 0.01 cD
**PM+AF**	0.72 ± 0.02 aA	0.70 ± 0.01 bA	0.59 ± 0.02 cB	0.59 ± 0.01 cB	0.58± 0.01 cB	0.46 ± 0.02 cC	0.33 ± 0.02 dD

Table S4. Temporal variations in lignin monomer ratio (G-lignin / S-lignin) in the degradation residues during the 15-day experiments at 2-day intervals. Experiments: Control, uninoculated control; PM, *Pseudomonas mandelii*; AF, *Aspergillus fumigatus*; PM+AF, co-culture of the two microbes. Different lower- and uppercase letters represent a significant difference ($P < 0.05$) at different experiments and stages.

Experiment	δ^2H_{LM} values of lignin methoxy in the degradation residues (mUr)						
	**3 days**	**5 days**	**7 days**	**9 days**	**11 days**	**13 days**	**15 days**
**Control**	-232.0 ± 1.9 bA	-236.7 ± 1.3 cB	-232.7 ± 1.1 bAB	-234.3 ± 1.2 bAB	-231.4 ± 0.6 abA	-233.0 ± 1.9 bAB	-230.4 ± 1.9 aA
**PM**	-229.1 ± 2.0 aAB	-227.3 ± 0.9 bA	-231.5 ± 1.2 bB	-232.4 ± 1.5 abB	-233.2 ± 1.2 bB	-231.9 ± 0.3 bB	-230.0 ± 2.3 aAB
**AF**	-226.3 ± 0.9 aA	-224.8 ± 1.3 abA	-226.6 ± 1.7 aA	-231.9 ± 1.3 abB	-228.4 ± 2.4 aAB	-226.4 ± 0.6 aA	-227.5 ± 2.2 aAB
**PM+AF**	-228.1 ± 1.0 aB	-223.8 ± 1.4 aA	-226.5 ± 1.6 aAB	-230.8 ± 1.1 aB	-228.3± 2.2 aB	-227.4 ± 0.8 aAB	-228.1 ± 1.1 aB

Table S5. The δ^2H_{LM} values of lignin methoxy in the degradation residues during the 15-day experiments at 2-day intervals. Experiments: Control, uninoculated control; PM, *Pseudomonas mandelii*; AF, *Aspergillus fumigatus*; PM+AF, co-culture of the two microbes. Different lower- and uppercase letters represent a significant difference ($P < 0.05$) at different experiments and stages.

Supplemental Text S1.

Details of the sample preparation and measurements for lignin monomers

1.1 Lignin monomer composition

We qualitatively and quantitatively analyzed the lignin monomers by means of gas chromatograph mass spectrometer (GC-MS) after the thioacidolysis reaction (Harman-Ware *et al.*, 2016). Specifically, we began by adding the following thioacidolysis reagents (3 mL) to analytical samples (15.0 ± 0.5 mg) in crimped glass vials (6 mL) with a polytetrafluoroethylene (PTFE)-lined screw-cap. 100 mL of fresh thioacidolysis reagent was prepared by adding 10 mL ethanethiol (EtSH), 2.5 mL boron trifluoride diethyl etherate (BF_3-$O(Et)_2$), and 87.5 mL dioxane. Next, the reaction vial was capped tightly and keep in a heating block for 4 h at 100 °C. After cooling in an ice-water bath (-20 °C) for 5 min, and added 0.2 mL of 1 mg/mL internal standard tetracosane ($C_{24}H_{50}$, dissolved in methylene chloride (CH_2Cl_2)) into the reaction mixture. We then neutralized the solution in a 10-mL separatory funnel, of which 0.25 to 0.30 mL of 0.4 mol/L sodium bicarbonate ($NaHCO_3$, dissolved in deionized water) was added to adjust the pH value to 3.5, followed by adding deionized water and extract the lignin monomer derivatives with CH_2Cl_2 three times. The upper and darker organic phase (CH_2Cl_2 solution) was transferred into a new glass vial (6 mL) by pipette. The combined organic solutions were dried over anhydrous magnesium sulfate ($MgSO_4$), and evaporated under reduced pressure at 45°C. The residue was dissolved in CH_2Cl_2 (0.3 mL) and derivatized by adding 35 µL pyridine (C_5H_5N, 98% v/v) and 75 µL N,O-bis(trimethylsilyl)acetamide ($C_8H_{21}NOSi_2$, 95% v/v), and letting the solutions sit for 4 h at room temperature (25 °C). Before injection for GC-MS analysis, the derivative solutions to be measured should been passed through hydrophobic membranes (0.22 µm) to filter out any impurities.

The measurements were obtained using a 7890B-7000D GC-MS (Agilent, USA) configured with a G4513A auto-injector (Agilent, USA) at the Key Laboratory of Soil Resource & Biotech Applications, Shaanxi Academy of Sciences. The gas chromatograph was fitted with an HP-5MS column (30 m × 0.32 mm × 0.25 μm; Agilent, USA). The following GC conditions were used: inlet temperature 250 °C, injection volume 1 μL, split injection (10:1), initial oven temperature at 150 °C for 2 min, ramp at 20 °C/min to 250 °C and held for 5 min. Helium were used as carrier gas at constant flow of 1.0 mL/min. The following MS conditions were used: electron ionization (EI) mode, ion source temperature 230 °C, interface temperature 250 °C, electron energy 70 eV, solvent delay 3.5 min, scan range from 40 to 650 amu. The GC-MS was run in selective ion monitoring mode for the following molecular ions and retention times: m/z 269 for G-lignin derivant at 17.5 min, m/z 299 for the S-lignin derivant at 20.8 min, and m/z 338 for internal standard tetracosane at 12.7 min.

The final determination of lignin monomer content was followed the method of Lapierre et al. (1986), the response factor (k) was the ratio of the relative concentration to the relative area between the internal standard and the target sample:

$$k = (C_s/C_i) \, / \, (A_s/A_i)$$

where k was equal to 1.5, C_s and C_i represent the concentrations of the lignin monomer derivative and the tetracosane, respectively, and A_s and A_i represent the corresponding peak areas, respectively. Moreover, we defaulted that the thioacidolysis reaction was sufficient and complete in this study, and defined the conversion of substrate and the recovery of internal standard to be 100% (Robinson and Mansfield, 2009; Wu et al., 2016).

1.2 Lignin methoxy groups stable hydrogen isotope ratio (δ^2H_{LM} values)

δ^2H_{LM} values of analytical powdered samples were measured as headspace iodomethane (CH_3I), released upon the selective substitution reaction between the methoxy groups of the degradation residues and hydriodic acid (HI). We followed the established method of Keppler et al. (2007) and Greule et al. (2008) with minor modifications (Lu et al., 2020). Specifically, HI (0.5 mL; 55-58% aqueous solution, Macklin, Shanghai, China) was added to degradation samples (10 ± 0.5 mg) in a brown crimp glass vial (1.5 mL; Agilent, USA) containing a tiny magneton. The vial was sealed with aluminum caps containing PTFE lined butyl rubber septa (11 mm crimp and thickness 0.9 mm) and stirred in oil bath at 120 °C for 30 min. This conversion temperature was a weighted value based on validation by Keppler et al. (2007) at 110 °C and Greule et al. (2008) at 130 °C. After incubating, the sub-samples were followed to equilibrated at 22 ± 0.5 °C (air-conditioned room) for at least 40 min. Finally, an aliquot (80 - 100 μL) of the CH_3I was directly injected into the analytical system by using a manual gas-tight syringe (100 μL; Hamilton, Reno, USA).

δ^2H_{LM} values of CH_3I were measured using a TRACE 1310 gas chromatograph (GC), coupled with an ISOLINK II Delta V Advantage isotope-ratio mass spectrometer (IRMS) via a thermal conversion reactor (ceramic tube [Al_2O_3], length 320 mm, 0.5 mm i.d., reactor temperature 1400 °C) (Thermo Fisher, Germany). The measurements were performed at the laboratory of biogeochemistry, Shaanxi Normal University. The GC was fitted with a TG-5MS column (30 m × 0.25 mm × 0.25 μm; Thermo Fisher, Germany), and based on the following parameters: inlet temperature 200 °C, split injection (12:1), initial oven temperature at 40 °C for 3.8 min, ramp at 20 °C/min to 80 °C and holding for 1 min, and then ramp at 40 °C/min to a final temperature of 100 °C and hold for 3 min. Helium was used as the carrier gas at a constant flow of 0.8 mL/min. High purity hydrogen gas (99.999%; Beijing AP Baif

Gases Industry Co., China) were used as the monitoring gas. The H_3^+ factor ranged from 4.7 to 5.0 ppm/nA during the measurement period.

Due to the lack of available commercial samples with true δ^2H_{LM} values as reference materials, two homogenized wood samples (*Larix gmelinii* and *Cryptomeria fortunei Hooibrenk*, shorted for LG and CFH) as in-house standards to assess the reproducibility of the isotope measurements and to calibrate the analytical accuracy of target samples. These two homogenized woods were collected from Northeastern (permafrost area) and Southwest (Sichuan Basin) China, and their δ^2H_{LM} values ranged a wide span (LG: -307.5 ± 2.3 mUr and CFH: -210.1 ± 2.5 mUr), which have been recently measured by the stable isotope lab of Frank Keppler's research group at the Heidelberg University against the methoxy group standards HUBG2&3 (Greule *et al.*, 2020; 2021). Whereas in our lab, the difference of the two measurements of one sample less equal to 3 mUr was defined as normal values, otherwise it would be measured again. The authors are aware that reliable stable isotope measurements ideally require well calibrated reference materials enabling a two-point normalization of raw δ-values. However, all measured δ^2H_{LM} values in this study were reported relative to the monitoring hydrogen gas and calibrated against our in-house standards. Moreover, some uncertainties could arise from external reaction, baseline drift, standard deviation, calibration procedure, and scale compression (Debajyoti *et al.*, 2010; Lee *et al.*, 2019; Greule *et al.*, 2020), nevertheless, because we analyzed relative differences in different experiments the reported results are allowed.

Supplemental References

Debajyoti, P., Grzegorz, S., István, F., 2010. Normalization of measured stable isotopic compositions to isotope reference scales – a review. *Rapid Communications in Mass Spectrometry*, 21, 3006-3014. doi: 10.1002/rcm.3185

Greule, M., Mosandl, A., Hamilton, J.T.G., Keppler, F., 2008. A rapid and precise method for determination of D/H ratios of plant methoxyl groups. *Rapid Communications in Mass Spectrometry*, 22, 3983-3988. doi: 10.1002/rcm.3817

Greule, M., Moossen, H., Lloyd, M.K., Geilmann, H., Brand, W.A., Eiler, J.M., Qi, H.P., Keppler, F., 2020. Three wood isotopic reference materials for δ^2H and δ^{13}C measurements of plant methoxy groups. *Chemical Geology*, 533, 119428. doi: 10.1016/j.chemgeo.2019.119428

Greule, M., Wieland, A., Keppler, F. (2021) Measurements and applications of δ^2H values of wood lignin methoxy groups for paleoclimatic studies. *Quaternary Science Reviews*, 268, 107107. doi: 10.1016/j.quascirev.2021.107107

Harman-Ware, A.E., Foster, C., Happs, R.M., Doeppke, C., Meunier, K., Gehan, J., Yue, F.X., Lu, F.C., Davis, M.F., 2016. Quantitative analysis of lignin monomers by a thioacidolysis method tailored for higher-throughput analysis. *Journal of Biotechnology*, 11, 1268-1273. doi:10.1002/biot.201600266

Keppler, F., Harper, D.B., Kalin, R.M, Meier-Augenstein, W., Farmer, N., Davis, S., Schmidt, H.L., Brown, D.M., 2007. Stable hydrogen isotope ratios of lignin methoxyl groups as a paleoclimate proxy and constraint of the geographical origin of wood. *New Phytologist*, 176, 600-609. doi: 10.1111/j.1469-8137.2007.02213.x.

Lapierre, C., Monties, B., Rolando, C., 1986. Thioacidolysis of poplar lignins: identification of monomeric syringyl products and characterization of guaiacyl-syringyl lignin fractions. *Holzforschung*, 40, 113-118. doi: 10.1515/hfsg.1986.40.2.113

Lee, H., Feng, X.J., Mastalerz, M., Feakins, S.J., 2019. Characterizing lignin: Combining lignin phenol, methoxy quantification, and dual stable carbon and hydrogen isotopic techniques. *Organic Geochemistry*, 136, 103894. doi:

10.1016/j.orggeochem.2019.07.003

Lu, Q.Q., Liu, X.H., Anhäuser, T., Keppler, F., Wang, Y.B., Zeng, X.M., Zhang, Q.L., Zhang, L.N., Wang, K.Y., Zhang, Y., 2020. Tree-ring lignin proxies in *Larix gmelinii* forest growing in a permafrost area of northeastern China: temporal variation and potential for climate reconstructions. *Ecological Indicators*, 118, 106750. doi: 10.1016/j.ecolind.2020.106750

Robinson, A. R., Mansfield, S. D., 2009. Rapid analysis of poplar lignin monomer composition by a streamlined thioacidolysis procedure and near-infrared reflectance-based prediction modeling. *Plant Journal*, 58, 706-714. doi: 10.1111/j.1365-313X.2009.03808.x

Wu, L., Rencoret, J., Lu, F. C., Karlen, S. D., Smith, B. G., Harris, P. J., del Río, J. C., Ralph, J., 2016. Tricin-lignins: occurrence and quantitation of tricin in relation to phylogeny. *Plant Journal*, 88, 1045-1057. doi: 10.1111/tpj.13315
